# Effect of Marine Red Yeast Rhodosporidium paludigenum on Diarrhea Rate, Serum Antioxidant Competence, Intestinal Immune Capacity, and Microflora Structure in Early-Weaned Lambs

**DOI:** 10.1155/2022/2228632

**Published:** 2022-04-15

**Authors:** Mengjian Liu, Wujun Liu, Wenju Zhang, Junli Niu, Jun Yao

**Affiliations:** ^1^Shihezi University, Shihezi, China; ^2^Xinjiang Agricultural University, Wulumuqi, China

## Abstract

In this study, the influences of marine red yeast Rhodosporidium paludigenum (MRYP) on diarrhea rate, serum antioxidant capacity, intestinal immunity capacity, and microflora structure of early-weaned lamb were investigated in a 60-day feeding trial. A total of 96 early-weaned lambs were utilized in this study. The lambs were divided into four experimental groups based on the percentage of marine red yeast Rhodosporidium paludigenum (MRYP) as milk replacer supplement. The rates of milk replacer supplement for the four groups were 0, 0.1%, 0.3%, and 0.5% of marine red yeast Rhodosporidium paludigenum (MRYP), respectively. The study was continued for 30 days. The results showed that (1) compared with control group, 0.5% marine red yeast Rhodosporidium paludigenum (MRYP) supplementation caused significantly decreases in average fecal score and diarrhea frequency by 33.74% and 40.23% (*P* < 0.05). (2) No significant difference was found in all tested related antioxidant indexes in serum of four treatments (*P* > 0.05). (3) The concentrations of SIgA, IgG, and IL-10 of group IV was significantly increased by 17.78%, 18.27%, and 8.17%, but the IL-6 and TNF-*α* were significantly decreased by 21.20% and 31.80%, compared to group I in the colon (*P* < 0.05). (4) The number of *Bifidobacterial* and *Lactobacilli* of group IV was significantly increased by 14.87% and 15.09%, but *Escherichia coli* and *Salmonella* were significantly decreased by 20.19% and 10.15%, compared to group I in the colon (*P* < 0.05). (5) A portion of marine red yeast Rhodosporidium paludigenum (MRYP) survived in the intestine of early-weaned lamb, and the number of survival marine red yeast Rhodosporidium paludigenum (MRYP) increased as the addition of marine red yeast Rhodosporidium paludigenum (MRYP) increased from 0.1 to 0.5% in milk replacer. Therefore, marine red yeast Rhodosporidium paludigenum (MRYP) has a potential to be a replacer of antibiotics for prevention and treatment of diarrhea in early-weaned lambs.

## 1. Introduction

Early weaning shortens the breeding cycle of ewes, which improves the flock productivity by increasing the frequency of lambing and is thus a common practice in modern sheep farming [1]. However, the digestive and immune system of early-weaned lambs were immature; that was when they had to adapt the drastic change from digestible watery milk to a less digestible solid feed. Therefore, the risks of enteric diseases, diarrhea, and infection by pathogenic bacteria, such as *Salmonella* and *Escherichia coli*, were increased [2–5]. Moreover, the early-weaned lambs are suffering from insufficient nutrient utilization, compromised immune system, and imbalance in intestinal homoeostasis during the abrupt weaning practices [6–9]. So the increased occurrences of diarrhea and mortality always happen in early-weaned lambs in local pastures (Changji, Xinjiang, China). Antibiotics as a supplement in feed were widely used to overcome the weaning stress syndrome and disruption of growth performance in early-weaned lambs [10]. But an exhaustive reliance on antibiotics has led to increase in risks of drug residues and antimicrobial resistance [11]. The balance of intestinal flora and the immune system in lambs might be destroyed by antibiotics, and the overprescribed antibiotic residual could be transmitted to humans ultimately [12]. Therefore, consistent efforts have been put in to find suitable natural substitutes for antibiotics to achieve pollution-free and on-environmental damage breeding in sheep farming.

Marine red yeast Rhodosporidium paludigenum (MRYP) is a kind of aquatic eukaryote, which is widely distributed in various ocean areas, deep seas, and lakes [13]. Some reports have indicated that MRYP have many superior characteristics. First of all, compared with terrestrial yeasts, marine yeasts have the potential for functional biomolecules by living in the extreme environments [14]. Other reports showed that MRYP has higher tolerant of high temperature, high pressure, high acidic, and oxygen-deficient environment, which indicated that MRYP may have a possibility of surviving in the rumen and intestine of ruminant [15]. Besides, MRYP lives in a unique environment with low light, low nutrition, and high salinity, which indicate that the metabolism and products of MRYP may be different from terrestrial yeasts. Besides, it was said that MRYP could secrete kinds of active metabolites, such as carotenoids, polysaccharides, unsaturated fatty acids, astaxanthin, and essential amino acids, which have high biological functions and nutritional value in animals [16]. Recently, marine red yeast has been widely used in aquaculture, and the biological functions, such as antioxidant competence, immunoenhancement activity, and probiotic, have been found in many studies [17]. However, it keeps little knowledge about the effects of MRYP on mammals, especially on early-weaned lambs. In an era of antibiotic resistance, we infer that MRYP might have a huge potential in the sheep farming.

In this experiment, we evaluated the effect of MRYP on average fecal score and diarrhea frequency. Besides, we tested lipids to assess oxidative stress status and oxidative damage of proteins. In addition, we also evaluate the effects of MRYP on immune indexes and microflora structure of the colon. It is hypothesized that the supplementation of MRYP in milk replacer will improve intestinal health indices and has the potential to become a candidate of substitutes for antibiotic in early-weaned lambs.

## 2. Materials and Methods

All experiments and animals followed the Shihezi University guidelines (case number: FRI-A2019-156-01). The experiment was conducted in Asar farming cooperatives, Changji, China (43°91′22.01^″^ S, 87°09′93.84^″^ W). The experiment site is characterized by an average elevation of approximately 2000 m and an average annual temperature of -10°C.

### 2.1. Preparation of MRYP

The experimental yeast strain was collected from Yarkand River, Kashi, China, and isolated with the YEPD medium and identified by 16S rRNA multiple sequence alignment. The marine red yeast was multiplied and obtained through the processes of fermentation, extraction, and purification which followed the conditions in previous study [14]. The MRYP of nutritional compositions were polysaccharides (≥84%), crude protein (≤4%), crude fat (≤3%), crude ash (≤2%), and moisture (≤7%).

### 2.2. Animal, Diets, and Experiment Design

Ninety-six crossbred Suffolk♂ ∗Altay♀ ∗Kazak♂ early-weaned lambs (7.51 ± 0.69 kg) were assigned randomly to 4 dietary treatments with 3 replicates (*n* = 8 animals/replicate). According to the 8-25 kg lambs with the daily gain 200 g/day of the Chinese Feeding Standard, the milk replacer was formulated as Table 1. Four groups of lambs were fed, respectively: group I, milk replacer without supplement; group II, milk replacer with 0.1% MRYP (0.84 g pure MRYP was added in 1.0 kg milk replacer); group III, milk replacer with 0.3% MRYP (2.52 g pure MRYP was added in 1.0 kg milk replacer); and group IV, milk replacer with 0.5% MRYP (4.20 g pure MRYP was added in 1.0 kg milk replacer).

All lambs were forcedly weaned at 35 days old and housed in pens (2.5 × 2.0 m) which were equipped with heating facilities (maintained at 22°C inside) and straw paillasses (thickness 2 cm). Milk replacer and warm water were available ad libitum in a whole feeding trial. Feeding and vaccination programs were followed as scheduled by the farm management. The milk replacer was mixed with warm water (50°C) with the ratio (milk replacer/water = 1 : 2), and 4 feeding times were arranged each day at 8:00, 13:00, 18:00, and 23:00.

### 2.3. Sample Collection

On the morning of the day 30, the 4 groups of blood samples were randomly filled into 20 mL tubes (with 0.1 mL 0.04% EDTA anticoagulants) by caudal venipuncture from four lambs (three males and one female) in each pen. Each tube of blood sample was centrifuged for 15 min at 3000 r/min at 4°C, and serum was stored at −80°C for carrying out the measurements of antioxidant activity analysis [18].

After the blood samples were collected, those four lambs in each pen were anesthetized by injecting of 4% Nembutal solution. After 30 min injection, the lambs were sacrificed by cutting the neck veins. After dissection, the colon segments of lambs were put into aseptic bags for the following immune analysis. The other colon segments with content were put into aseptic boxes for the following bacteriological examination. After that, all samples were stored in a liquid nitrogen jar.

### 2.4. The Effect of MRYP on Diarrhea Incidence

As shown in Table 2, a hierarchical system of average fecal score was used to imply the severity extent and presence of diarrhea. The presence and severity extent of diarrhea were applied by a hierarchical system, as shown in Table 2. During the whole experiment, if a fecal score is higher than 3 for 3 consecutive days, it would be considered diarrheic [19]. After the experiment, the average fecal score and diarrhea frequency would be calculated by using the following formulas: Average fecal score (%) = Sum of fecal scores/the number of lambs and Diarrhea frequency (%) = (the number of diarrhea lambs × diarrhea days)/(the total number of lambs × experimental days)∗100%.

### 2.5. Determination of Antioxidant Indexes

#### 2.5.1. Estimation of Catalase (CAT)

The sample (0.1 mL) was added in H_2_O_2_ (0.4 mL), phosphate buffer (0.9 mL), and dichromate-acetic acid (1.0 mL). After that, the mixture was boiled in boiling water bath for 10 min. The color was observed at 620 nm spectrophotometrical analysis. The blank containing reagent alone and 1.2–6.0 *μ*mol of the standards were prepared. And the CAT was manifested in *μ*mol/mL [20].

#### 2.5.2. Estimation of Superoxide Dismutase (SOD)

The sample (1.0 mL) was added to water (2.0 mL), ethanol (2.5 mL), and chloroform (1.5 mL). The mixture was centrifuged in shaking condition for 90 min. The SOD assay proceeds by adding sodium pyrophosphate buffer (1.2 mL), enzyme (1.4 mL), phenazinemethosulfateate (0.1 mL), nitroblue tetrazolium (0.3 mL), and 0.2 mL nicotinamide adenine dinucleotide (NADH). The mixture was incubated at 30°C for 100 min and the reaction was terminated by adding 1.0 mL glacial acetic acid. The 4 mL of n-butanol was added in the mixture and allowed to stand for 10 mins. After that, the n-butanol layer was separated and the color of the n-butanol chromogen was detected spectrophotometrically at 520 nm. The standards were prepared in the same way. The SOD activity was expressed as unit/mL [21].

#### 2.5.3. Estimation of Reduced Glutathione (GSH)

The sample (2.0 mL) was added in the disodium hydrogen phosphate (4.0 mL) and Ellman's reagent (1.0 mL). The color was measured at 412 nm in spectrophotometer. The blank was prepared with 1.0 mL of buffer. The GSH activity was expressed as *μ*mol/mg [22].

#### 2.5.4. Estimation of MDA (Malondialdehyde)

The sample (1.0 mL) was added in 1.0 mL of water and 1.5 mL of 0.67% thiobarbituric acid (TBA). The color was measured at 535 nm spectrophotometrically. The results were expressed nmol/mL [23].

### 2.6. Determination of Immune Parameters

After the samples defrosted, 1.0 g colon segment was accurately weighed and grounded with liquid nitrogen in sterile mortars for 15 min. The colon segment powder was mixed with 5 mL 0.9% sodium chloride solution and centrifuged at 4°C, 3000 r/min, for 15 min, and then, the tissue fragments were removed, and the supernatant was collected [24]. The levels of SIgA, IgA, IgG, IL-6, IL-10, IFN-*γ*, and TNF-*α* in the colon were determined by the instruction of ELISA kits.

### 2.7. Determination of Intestinal Microflora

All procedures were implemented under sterile condition. The samples were defrosted, and 5 g of colon segments with contents was weighed. The numbers of the tested bacteria were detected according to the plate dilution counting (Table 3).

### 2.8. Statistical Analysis

The original data were counted and calculated preliminarily by EXCEL (Microsoft office version 2019 for Windows, Microsoft Inc., Chicago, IL, USA). The statistical values and significant differences among the means were statistically analyzed and represented as mean ± standard deviation by using one-way analysis of variance (ANOVA) by Duncan's test of SPSS software (version 18.0 for Windows, SPSS Inc., New York, IL, USA).

## 3. Results

### 3.1. The Effect of MRYP on Diarrhea Incidence

As shown in Table 4. Compared with group I, the average fecal scores of group III and group IV were significantly decreased by 33.74% and 31.30%, respectively (*P* < 0.05). The diarrhea frequency of group III and group IV was significantly lower than group I by 37.51% and 40.23% (*P* < 0.05).

### 3.2. The Effect of MRYP on Antioxidant Capacities

As shown in Table 5, there were no significant statistical differences in all experimental groups concerning the antioxidant activity of CAT, SOD GSH-PX, and MAD in serum (*P* > 0.05). However, the results show that the capacities of CAT, SOD, and GSH-PX of group IV were enhanced by 0.42%, 4.70%, and 2.38%, compared with group I. Besides, the content of MAD of group IV was decreased by 10.1% as compared with group I (*P* > 0.05).

### 3.3. The Effect of MRYP on Immune Responses in Colon

As shown in Table 6. Compared with group I, the content of SIgA, IgG, and IL-10 of group IV was significantly increased by 17.78%, 18.27%, and 8.17% (*P* < 0.05). Besides, the content of IL-6 and TNF-*α* of group IV was significantly decreased than group I by 21.20% and 31.80% (*P* < 0.05). However, the content of IgA and IFN-*γ* was not statistically different among the treatments (*P* > 0.05).

### 3.4. The Effect of MRYP on Intestinal Microflora in the Colon

Besides *Perfringocin*, all detected number of bacteria were statistically different among the treatments (*P* < 0.05) as shown in Table 7. Compared with group I, the number of *Bifidobacterium* and *Lactobacillus* of group IV was significantly increased by 14.87% and 15.09% (*P* < 0.05). The number of *Escherichia coli* and *Salmonella* of group IV was significantly decreased by 20.19% and 10.15% (*P* < 0.05). The MRYP has been, respectively, detected in groups II, II, and IV, and the number of survival MRYP increased as the addition of MRYP increased from 0.1 to 0.5% in milk replacer. Besides, the number of MRYP of group III and group IV was significantly higher than in group II (*P* < 0.05), respectively.

## 4. Discussion

Diarrhea is an intuitive implication of morbidity in early-weaned lambs feeding ([30]). According to statistics, a diarrheal lamb is more than twice as likely to die as normal one [31]. During the period of forced weaned, lambs have to adapt to the artificial feeding when their immune defense and digestive system were immature; therefore, the early-weaned lambs have to adapt restricted amounts of less digestible artificial solid feed [32, 33]. Kinds of enteric pathogens are recognized as one of the major reason, which have been implicated in lamb diarrhea [34]. Number of enteric pathogens can cause excess probiotics death, disrupting the intestinal flora balance and causing overreaction of the intestinal immune system and stress reaction. In this study, the MRYP supplement of 0.5% significantly decreased the average fecal scores and the diarrhea frequency. The main biological components of MRYP are yeast wall polysaccharides, which could decrease the diarrhea rate. First of all, some researches have proved that yeast wall polysaccharides could improve intestinal microbiota. It has been reported that the Pichia guilliermondii cell polysaccharides significantly decreased the number of pathogenic in the intestinal of chickens [35]. Zhang et al. found that yeast polysaccharide could decreased the rate of diarrhea in weaned piglets, caused by the number of Escherichia coli and Salmonella in the caecum of weanling piglets which was significantly reduced by yeast polysaccharide [36]. The addition of yeast wall polysaccharides significantly increased the alpha diversity of intestinal microbial in calf rumen [37]. Besides, some reports have proved that yeast wall polysaccharides could enhance immune system. Sheng F found that yeast polysaccharide significantly enhanced the level of the spleen index, serum IgA, IgG, and LZM. Another report showed that yeast polysaccharide had positive effect on the immunity of Apostichopus japonicus at normal states. Zhang et al. found that supplementation of yeast polysaccharide significantly increased the level of IgM in the serum of weaned piglets [36]. Therefore, adjusting the intestinal flora and enhancing the immune system may be the reason for decreasing the rate of diarrhea rate in lambs.

Excessive amount of reactive oxygen species will induce oxidative damage to organelles and finally injure functions of organs [38]. Such oxidative stress is an important causative factor for intestinal dysfunction, excessive immune, and diarrhea in lambs [39]. As what is well known, the levels of CAT, GSH-Px, and SOD play crucial roles in scavenging harmful reactive oxygen derivatives [40]. Those antioxidant enzymes ubiquitously exist in numerous cells and are extremely important for protecting the cell against the toxic products of aerobic respiration. MDA is associated with cytotoxic effects of H_2_O_2_. More decline of CAT, SOD activity, and GSH depletion indicate more MDA and cell damage [41]. In the present study, although the differences among all processed groups were not significant, the treatment with MRYP had a higher activity level of CAT, GSH-PX, and SOD and a lower level of MDA than the control group. This result indicated that MRYP has the possibility of antioxidant enhancing. Some yeast wall polysaccharides are structurally diverse class of large molecules, indicating that the inhibitory actions of excessive oxygen radical might be mediated by the surface binding of polysaccharides to cell-specific surface molecules. For example, it has been reported that the superoxide anion radical scavenging activity of polysaccharide appears to depend on the amount of peptides present in the form of polysaccharide–peptide complexes [42]. The other research found that the antioxidant mechanism may be due to the supply of hydrogen by yeast wall polysaccharides, which combines with radicals and forms a stable radical to terminate the radical chain reaction [43]. Besides, the antioxidant mechanism of yeast wall polysaccharides possibly is that yeast wall polysaccharides can combine with the radical ions which are necessary for radical chain reaction; then, the reaction is terminated [44]. However, the exact explanation of mechanism underlying the free radical scavenging activity exerted by yeast wall polysaccharides is still not fully understood.

The intestinal immune system is the first defense line against antigens, such as pathogenic microorganisms, viruses, and toxins. SIgA is a kind of major component of secretory immunoglobulin in gut mucosal immune, which could maintain the integrity of biological barrier, against the kinds of pathogenic bacteria, and decrease the severity and frequency of inflammatory responses in intestinal. IgA and IgG are primary antibodies in intestinal mucosa. TNF-*α*, IL-6, and IFN-*γ* are three important inflammatory cytokines in the intestinal immune system, and adaptive immune responses were activated for fighting against the pathogenic bacteria invasion. However, the excessive expression of TNF-*α*, IL-6, and IFN-*γ* in intestinal mucosal will cause an increasing permeability of blood vessels, which will make some damage of mucosal cellular. IL-10 could suppress the natural killer cells and inflammatory cytokine, and the immune tolerance would also be enhanced by IL-10. In this study, we proved that the addition of 0.5% MRYP enhanced the levels of IgG, SIgA, and IL-10, which indicated that the biological barrier and immune tolerance were enhanced. Besides, we found that the addition of 0.5% MRYP decreased the level of IL-6 and TNF-*α*, which suggested that the inflammatory response was suppressed. This finding might be a possible reason for relieving of diarrhea rate and weaned stress in early-weaned lambs.

It has been proved that intestinal microbial ecosystem and balance of intestinal flora structure is a virtual endocrine organ of the body [45]. There are frequent and sensitive interactions between the intestinal microbial communities in immune system. In this study, the addition of 0.5% MRYP significantly enhanced the number of *Lactobacillus* and *Bifidobacterium* but significantly suppressed the number of *Salmonella* and *Escherichia coli*, which presented that MRYP enhanced the balance of intestinal flora structure. The survival MRYP has been detected in experimental groups. In addition, the number of MRYP of group III and group IV was significantly increased than the control group. The result indicated that MRYP could survive in the intestine of early-weaned lamb. It has been reported that the addition of yeast extractive enhanced the number of *Bifidobacterium* but decreased *Escherichia coli* in the intestine of turkey [46]. It is reported that Saccharomyces cerevisiae significantly enhanced the number of *Bifidobacterium* and *Lactobacillus* but decreased *Salmonella* and *Escherichia coli* in the cecum of chicken [47].

## 5. Conclusions

To our best knowledge, this study provides the first evidence that MRYP can serve as an effective and beneficial feed additive for decreasing diarrhea rate of lambs, which may be partly attributed to the enhancement of colon immune function and probiotics, and for decreasing colon pernicious bacteria by MRYP. Besides, the antioxidant capacities in serum can be improved in a nonsignificant level by MRYP. As the results of diarrhea rate, antioxidant capacities, immune responses, and intestinal microflora, the optimum inclusion level of MRYP is 0.5% in feed replacer based on the current experimental condition.

## Figures and Tables

**Table 1:** Ingredient and mean chemical composition of milk replacer (air-dry basis) %.

Ingredients		Nutrition level	
Expended corn	41.50	Dry matter	90.67
Fermented soybean	20.00	Crude protein	23.75
Expended soy	16.00	Digestible energy DE (MJ/kg)	17.38
Baby formula milk powder	10.00	Crude fat	6.08
Alfalfa hay	8.00	Neutral detergent fiber	14.81
Soybean oil	2.00	Lysine	0.91
Premix	1.00	Threonine	0.65
CaHCO_3_	1.00	Methionine+cysteine	0.61
NaCl	0.30	Calcium	0.53
NaHCO_3_	0.20	Phosphorus	0.41

Without adding antibiotics in the ingredients, the nutrient levels were calculated. The per kilogram of premix supply: Zn, 30 mg; Mn, 20 mg; Fe, 15 mg; Cu, 4 mg; Se, 0.15 mg; I, 0.20 mg; Co, 0.08 mg; vitamin A, 9000 IU; vitamin B1, 2.8 mg; vitamin B2, 2.2 mg; vitamin B6, 6.6 mg; vitamin B12, 0.02 mg; vitamin D, 1000 IU; vitamin E, 28 IU; vitamin K, 0.08 mg; nicotinic acid, 10 mg; D-pantothenic acid, 8 mg; folic acid, 0.80 mg; biotin, 0.06 mg.

**Table 2:** A hierarchical system of average fecal score.

Score	Trait
1	Without fetid odor and hard firm feces
2	Without fetid odor but slightly soft feces
3	With a light fetid odor and soft, partially formed feces
4	With a distinct fetid odor and loose, semiliquid feces
5	With a pungent fetid odor and watery, mucous-like feces

**Table 3:** Culture medium and conditions of different tested bacteria.

Bacteria	Medium	Condition
*Lactobacillus*	MRS	42°C, pH = 6.3, anaerobic, 72 h [25]
*Bifidobacterium*	BBL	37°C, pH = 7.0, anaerobic, 48 h [26]
*Escherichia coli*	EMB	37°C, pH = 7.2, aerobiosis, 48 h [27]
*Salmonella*	HE	37°C, pH = 7.0, aerobiosis, 48 h [28]
*Clostridium perfringens*	SPS	37°C, pH = 7.0, anaerobic, 37°C, 48 h [29]

BBL: agar medium; MRS: Man, Rogosa, and Sharpe medium; EMB: eosin methylene blue agar; HE: Hektoen enteric agar; SPS: sulfite polymyxin sulfadizine agar.

**Table 4:** Effects of MRYP on diarrhea rate of early-weaned lambs.

Items	Group I	Group II	Group III	Group IV
Average fecal score	2.46 ± 0.11b	2.06 ± 0.19ab	1.69 ± 0.08a	1.63 ± 0.05a
Diarrhea frequency (%)	14.69	12.65	9.18	8.78

**Table 5:** Effects of MRYP on antioxidant capacity in serum of early-weaned lambs.

Items	Group I	Group II	Group III	Group IV
CAT (*μ*mol/mL)	16.53 ± 2.06	15.92 ± 3.32	16.64 ± 2.58	16.60 ± 1.37
SOD (U/mL)	1.70 ± 0.36	1.72 ± 0.56	1.74 ± 0.50	1.78 ± 0.17
GSH-PX (*μ*mol/L)	16.37 ± 0.21	16.44 ± 2.36	16.59 ± 2.12	16.76 ± 1.44
MDA (nmol/mL)	1.98 ± 0.33	1.94 ± 0.10	1.83 ± 0.35	1.78 ± 0.16

**Table 6:** Effects of MRYP on the content of antibody and cytokine in the colon membrane of lambs.

Items	Group I	Group II	Group III	Group IV
SIgA (mg/g)	14.62 ± 1.67a	15.90 ± 0.11b	16.74 ± 1.04c	17.22 ± 0.33c
IgA (mg/g)	30.05 ± 3.38	31.08 ± 5.85	31.97 ± 5.41	31.38 ± 3.75
IgG (mg/g)	39.62 ± 2.64a	39.81 ± 3.41a	42.55 ± 4.23b	46.86 ± 1.78b
IL-6 (pg/mL)	3.82 ± 0.27b	3.84 ± 0.14b	3.51 ± 0.58b	3.01 ± 0.35a
IL-10 (pg/mL)	4.04 ± 1.50a	3.42 ± 1.01a	4.28 ± 1.25ab	4.37 ± 0.84b
TNF-*α* (pg/mL)	4.56 ± 0.21b	4.20 ± 0.37b	3.27 ± 0.18a	3.11 ± 0.59a
IFN-*γ* (pg/mL)	3.09 ± 0.78	3.16 ± 0.13	2.95 ± 0.09	2.83 ± 0.44

**Table 7:** Effects of MRYP on the number of bacteria in the colon (log CFU/g).

Tested bacteria	Group I	Group II	Group III	Group IV
*Lactobacillus*	6.03 ± 0.53a	6.55 ± 0.36ab	6.61 ± 0.21b	6.94 ± 0.62b
*Bifidobacterium*	5.11 ± 0.14a	4.97 ± 0.02a	5.31 ± 0.32ab	5.87 ± 0.51b
*Escherichia coli*	4.26 ± 0.32b	4.14 ± 0.25b	3.98 ± 0.17ab	3.40 ± 0.40a
*Salmonella*	5.42 ± 0.15b	5.39 ± 0.73b	5.10 ± 0.14a	4.87 ± 0.36a
*Perfringocin*	5.29 ± 2.40	5.04 ± 0.15	4.91 ± 0.07	4.97 ± 0.18
*MRYP*	Undetected	1.25 ± 0.15a	3.88 ± 0.15b	4.16 ± 0.15b

## Data Availability

All data generated or analyzed during this study are included in this published article All primary data generated during and/or analyzed during the current study are available from the corresponding author on reasonable request.
